# Association of pericoronary adipose tissue inflammation with coronary collateral vessel formation in patients with chronic left anterior descending occlusion

**DOI:** 10.1093/ehjopen/oeaf155

**Published:** 2025-11-27

**Authors:** Chen Lv, Kang Zhou, Xing-Dou Chen, Qi-Ying Wu, Jin-Chang Li, Xian-Ming Fu

**Affiliations:** Department of Cardiovascular Surgery, The Second Xiangya Hospital, Central South University, No.139, Renmin Road Central, Changsha, Hunan 410011, P.R. China; Department of Cardiovascular Surgery, The Second Xiangya Hospital, Central South University, No.139, Renmin Road Central, Changsha, Hunan 410011, P.R. China; Department of Nuclear Medicine, The Second Xiangya Hospital, Central South University, No.139, Renmin Road Central, Changsha, Hunan 410011, P.R. China; Department of Cardiovascular Surgery, The Second Xiangya Hospital, Central South University, No.139, Renmin Road Central, Changsha, Hunan 410011, P.R. China; Department of Cardiovascular Surgery, The Second Xiangya Hospital, Central South University, No.139, Renmin Road Central, Changsha, Hunan 410011, P.R. China; Department of Cardiovascular Surgery, The Second Xiangya Hospital, Central South University, No.139, Renmin Road Central, Changsha, Hunan 410011, P.R. China

**Keywords:** Coronary collateral circulation, Pericoronary adipose tissue, Macrophage polarization, Angiogenesis, Coronary artery disease

## Abstract

**Aims:**

The role of pericoronary adipose tissue (PCAT) inflammation in coronary collateral circulation (CCC) development remains unclear. This study compared PCAT characteristics in chronic total occlusion (CTO) patients with good vs. poor collaterals.

**Methods and results:**

Twenty left anterior descending CTO patients were stratified into poor (P-CCC, *n* = 8) and good collateral (G-CCC, *n* = 12) groups per Rentrop classification. CT-derived fat attenuation index (FAI), epicardial adipose tissue (EAT) volume, and histologic markers (macrophage polarization, microvascular density) were analyzed. Compared with the P-CCC group, the G-CCC group exhibited significantly lower FAI (−93.6 ± 7.2 vs. −70.8 ± 2.4 HU, *P* < 0.05) but higher EAT volume [8491.3 (7951.6–13060.0) vs. 3452.8 (1741.7–6425.4) mm³, *P* < 0.05]. Histologically, relative to P-CCC PCAT, G-CCC PCAT showed increased M2 macrophage density (14.47 ± 2.87 vs. 3.47 ± 1.63, *P* < 0.05), a higher M2/M1 ratio (4.40 ± 2.17 vs. 0.95 ± 0.61, *P* < 0.05), and greater microvascular density (4.69 ± 1.11 vs. 2.21 ± 0.50, *P* < 0.05).

**Conclusion:**

PCAT inflammation is associated with enhanced collateral vessel formation. These findings highlight PCAT's potential as a therapeutic target for collateral promotion, warranting further investigation into its molecular mechanisms.

## Graphical Abstract

**Graphical Abstract:**
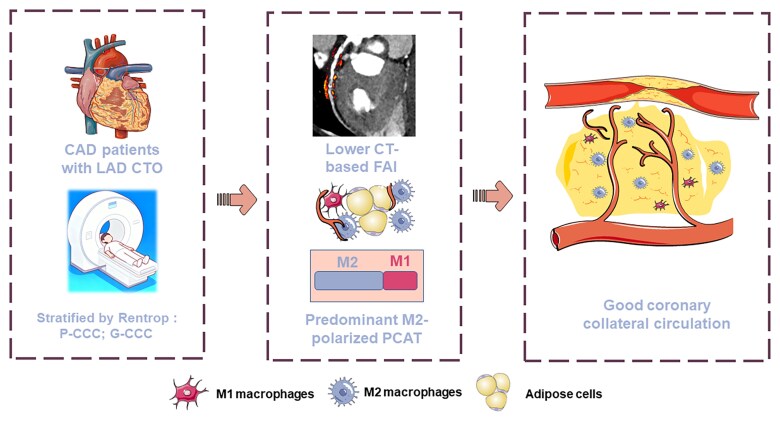

## Introduction

Coronary collateral circulation (CCC) is a critical compensatory mechanism in coronary artery disease (CAD).^1^ Pericoronary adipose tissue (PCAT), which directly contacts coronary vessels, secretes inflammatory mediators and contributes to CAD pathogenesis.^2^ Although EAT has been linked to CCC,^3–5^ the association between PCAT inflammation and CCC formation remains not well defined. Therefore, based on its anatomical proximity and paracrine activity, we propose that PCAT inflammation is directly associated with CCC formation in patients with chronic total occlusion (CTO).

## Materials and methods

**Figure 1:**
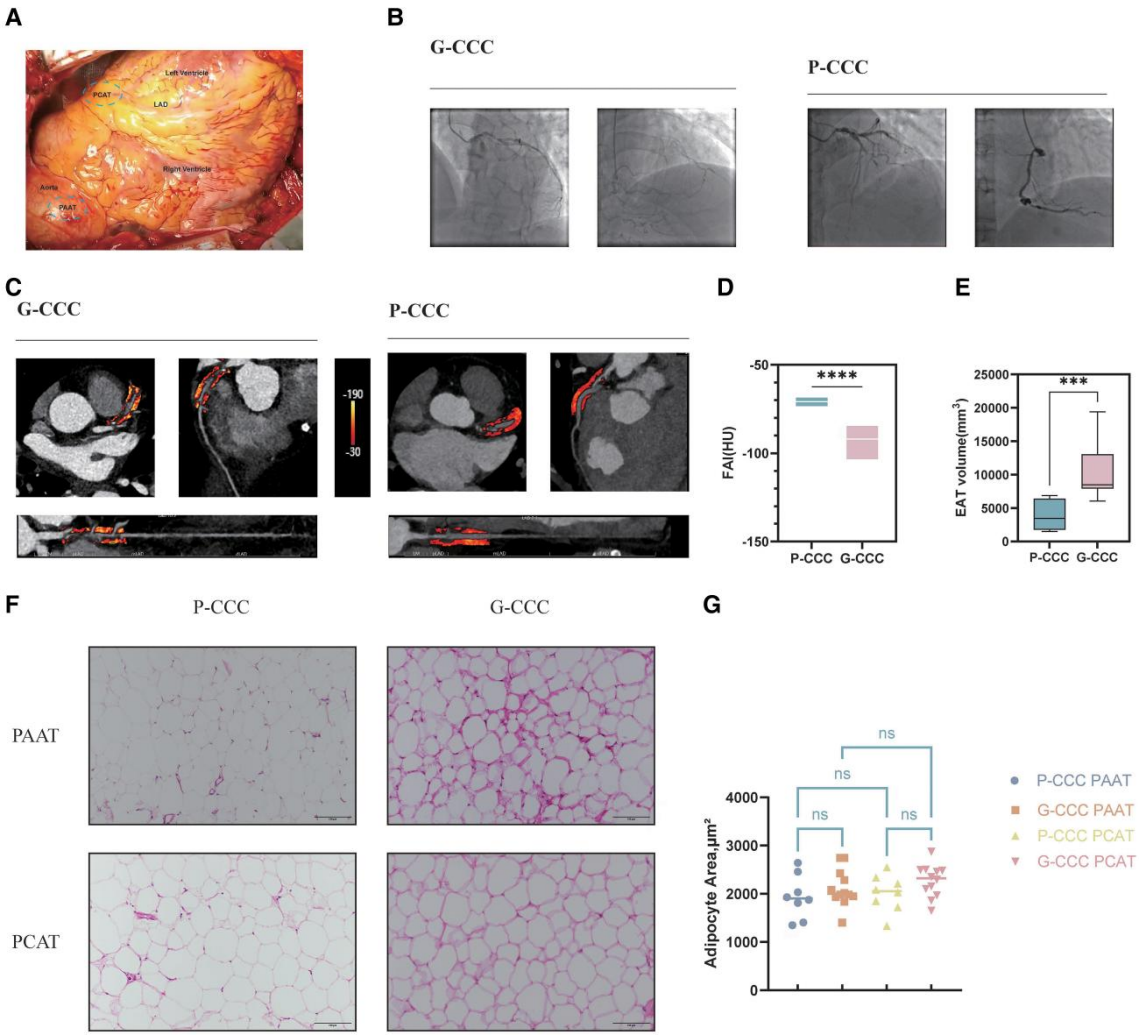
Imaging characteristics and adipocyte morphology of PCAT in patients with CAD. (*A*) Representative intraoperative views during CABG showing PCAT and PAAT around coronary arteries. Blue dotted lines indicate sampling areas. (*B*) Angiograms from G-CCC and P-CCC patients. P-CCC shows no collateral filling distal to LAD occlusion; G-CCC shows robust collateral supply from RCA. (*C*) Measurement of FAI along the proximal 40 mm of LAD on CT. Adipose voxels: −190 to −30 HU. (*D*) Comparison of FAI between P-CCC (*n* = 5) and G-CCC (*n* = 7) groups (*t*-test; mean ± SD). *P < 0.05, **P < 0.01, ***P < 0.001. (*E*) EAT volume in P-CCC (*n* = 8) and G-CCC (*n* = 12) groups (Mann–Whitney *U* test; median with IQR). *P < 0.05. (*F*) H&E staining of PAAT and PCAT in P-CCC and G-CCC groups. Scale bar: 100 μm. (*G*) Adipocyte area quantitative analysis of the P-CCC (*n* = 8) and G-CCC (*n* = 12) groups (one-way ANOVA; mean ± SD). *P < 0.05.

Twenty coronary artery bypass grafting candidates with severe stenosis three-vessel disease (≥70% stenosis) and/or left main CAD, complicated by left anterior descending artery (LAD) CTO, were enrolled. Preoperative coronary computed tomography angiography was performed to assess fat attenuation index (FAI) of LAD and EAT volume. Collateral circulation was graded per Rentrop.^3^ PCAT and PAAT samples obtained during surgery (*Figure 1A*) were analyzed using immunofluorescence for macrophage polarization (M1: iNOS⁺/CD68⁺; M2: CD206⁺/CD68⁺) and microvascular density (vWF⁺).

## Results

Baseline characteristics were comparable between groups (*Table 1*). G-CCC patients had significantly lower FAI (−93.6 ± 7.2 vs. −70.8 ± 2.4 HU, *P* < 0.05; *Figure 1C* and *D*) and higher EAT volume [8491.3 (7951.6–13060.0) vs. 3452.8 (1741.7–6425.4) mm³, *P* < 0.05; *Figure 1E*]. Adipocyte morphology showed no significant differences (*Figure 1F* and *G*).

**Table 1:** Baseline patient characteristics

Demographics	Total (*n* = 20)	P-CCC(*n* = 8)	G-CCC (*n* = 12)	*P* value^a^
Age, y	59.4 ± 9.3	59.6 ± 8.9	59.2 ± 10.0	0.918
Men	17.0 (85.0)	7.0 (87.5)	10.0 (83.3)	1.000
BMI, kg/m^2^	25.1 ± 3.0	25.4 ± 3.4	24.9 ± 2.9	0.684
Hypertension	17.0 (85.0)	7.0 (87.5)	10.0 (83.3)	1.000
Diabetes	9.0 (45.0)	5.0 (50.0)	5.0 (41.7)	1.000
Smoking	11.0 (55.0)	5.0 (62.5)	6.0 (50.0)	0.670
Prior PCI	4.0 (20.0)	1.0 (12.5)	3.0 (25.0)	0.619
Left Main Stenosis (≥50%)	4.0 (20.0)	1.0 (12.5)	3.0 (25.0)	0.619
LVEF, %	64.0 (46.3–68.8)	59.0 (43.0–67.8)	66.5 (46.3–69.8)	0.343
Laboratory data				
Total cholesterol, mmol/L	3.5 ± 0.9	3.4 ± 0.4	3.5 ± 1.1	0.758
LDL, mmol/L	2.0 ± 0.8	2.0 ± 0.4	2.0 ± 1.0	0.952
HDL, mmol/L	0.8 ± 0.1	0.8 ± 0.1	0.8 ± 0.1	0.802
Triglycerides, mmol/L	1.6 (1.2–2.3)	1.6 (1.3–2.3)	1.6 (1.0–2.1)	0.521
BNP, pg/mL	265.5 (69.8–603.9)	464.0 (123.7–2420.8)	252.0 (47.2–448.8)	0.305
Uric Acid, umol/L	369.3 ± 110.8	353.7 ± 83.1	379.7 ± 128.6	0.620
Blood Glucose, mmol/L	4.9 ± 1.5	4.3 ± 0.6	5.3 ± 1.8	0.156
HbA1c, %	6.7 ± 1.2	6.1 ± 0.5	7.0 ± 1.4	0.065
CRP, mg/L	1.2 (0.5–3.1)	1.1 (0.5–2.1)	1.4 (0.4–12.5)	0.624
Creatinine, μmol/L	101.5 (84.5–138.0)	126.5 (95.7–157.5)	89.5 (75.4–116.0)	0.082
WBC,10×9/L	6.8 ± 1.8	6.1 ± 1.3	7.3 ± 2.0	0.149
Neutrophil,10×9/L	4.2 ± 1.8	3.5 ± 0.9	4.7 ± 2.2	0.156
Lymphocyte,10×9/L	2.3 ± 1.9	1.9 ± 1.0	2.6 ± 2.3	0.420
MONO, %	7.2 ± 1.9	7.4 ± 2.2	7.2 ± 1.7	0.797
Haemoglobin, g/L	138.9 ± 17.3	136.9 ± 20.3	140.2 ± 15.8	0.681
Prehospital medications				
Statin	15.0 (75.0)	5 (62.5)	10 (83.3)	0.347
Beta blocker	12.0 (60.0)	4 (50.0)	8 (66.7)	0.648
Aspirin	15.0 (75.0)	5 (62.5)	10 (83.3)	0.347

Data shown are mean ± standard deviation (SD), median (interquartile range), or *n* (%).

BMI, Body mass index; BNP, B-type Natriuretic Peptide; CRP, C-Reactive Protein; HbA1c, Glycosylated Haemoglobin Type A1c; HDL, High-density lipoprotein; LDL, Low-density lipoprotein; LVEF, Left ventricular ejection fraction; MONO, monocytes; PCI, percutaneous coronary intervention; WBC, White Blood Cell.

^a^ *P* value comparing those with P-CCC and G-CCC.

**Figure 2:**
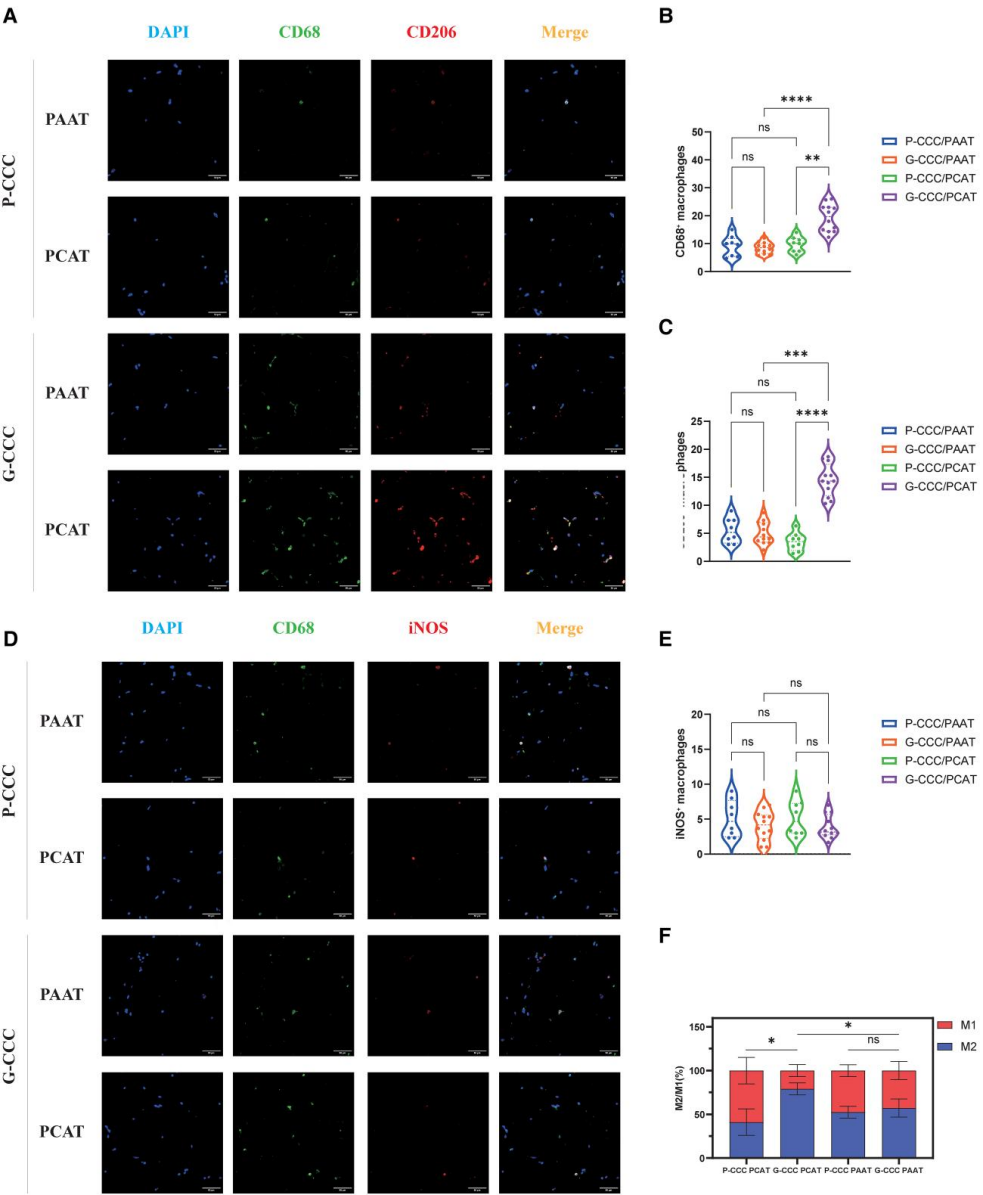
M2 macrophage polarization in PCAT of G-CCC patients. (*A*) Immunofluorescence for CD68, CD206, and DAPI (nuclei)) across groups. Scale bar: 50 μm. (*B*) CD68⁺ macrophage density (Kruskal-Wallis test; mean ± SD). *P < 0.05, **P < 0.01, ***P < 0.001. (*C*) CD68⁺CD206⁺ M2 macrophage density (Kruskal-Wallis test; mean ± SD). *P < 0.05, **P < 0.01, ***P < 0.001. (*C*) Immunofluorescence for CD68, iNOS, and DAPI (nuclei). Scale bar: 50 μm. (*D*) CD68⁺iNOS⁺ M1 macrophage density (one-way ANOVA; mean ± SD). (*E*) M1 and M2 proportions among CD68⁺ cells (Mann–Whitney *U* test; mean ± SD). *P < 0.05, **P < 0.01, ***P < 0.001.

The G-CCC group exhibited higher CD68⁺ macrophage density in PCAT vs. P-CCC PCAT (19.94 ± 6.22 vs. 9.79 ± 2.72, *P* < 0.05; *Figure 2B*). M2 macrophages were markedly enriched in G-CCC PCAT (14.47 ± 2.87 vs. 3.47 ± 1.63, *P* < 0.05; *Figure 2C*), while M1 counts showed no significant difference (*Figure 2E*). The M2/M1 ratio was elevated in G-CCC PCAT (4.40 ± 2.17 vs. 0.95 ± 0.61, *P* < 0.05; *Figure 2F*).

**Figure 3:**
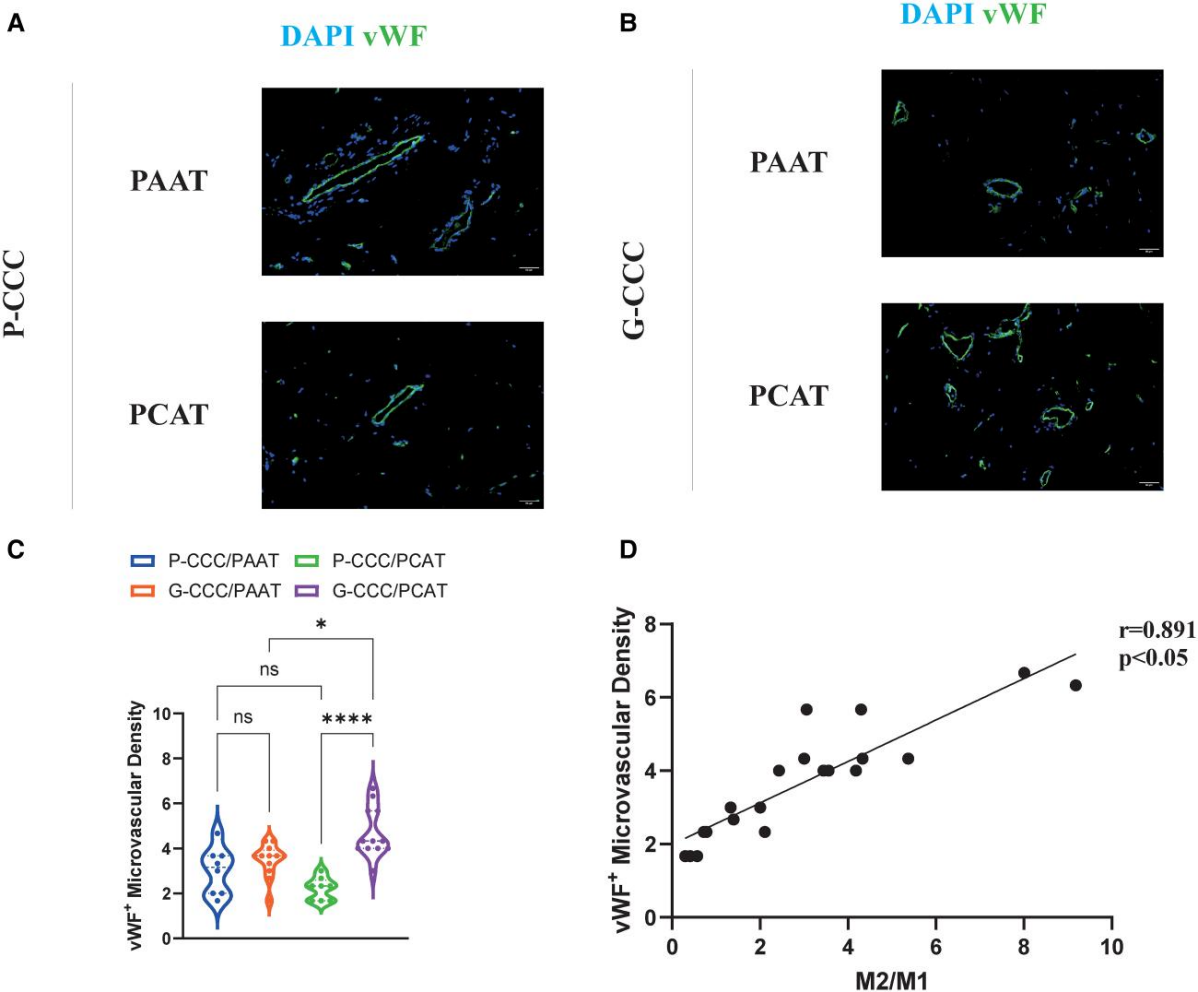
Increased microvessel density and pro-angiogenic adipokine profile in PCAT with good collateral circulation. (*A*) vWF⁺ vessels in P-CCC PCAT and PAAT. DAPI (nuclei). Scale bar: 50 μm. (*B*) vWF⁺ vessels in G-CCC tissues. Scale bar: 50 μm. (*C*) vWF⁺ microvessel density across groups (one-way ANOVA; mean ± SD). *P < 0.05, **P < 0.01, ***P < 0.001. (*D*) Correlation between M2/M1 ratio and vWF⁺ density (Pearson; *n* = 20).

Microvascular density was higher in G-CCC PCAT vs. P-CCC PCAT (4.69 ± 1.11 vs. 2.21 ± 0.50, *P* < 0.05; *Figure 3C*) and vs. G-CCC PAAT (4.69 ± 1.11 vs. 3.47 ± 0.74, *P* < 0.05). The M2/M1 ratio correlated strongly with micro vessel density (*Figure 3D*).

## Discussion

EAT and PCAT serve not only as energy reserves but also contribute to CAD progression.^2^ Although increased EAT thickness has been linked to collateral vessel presence,^3^ we specifically found lower FAI around the LAD in the G-CCC group vs. the P-CCC group. Importantly, since elevated FAI reflects pericoronary inflammation—which is associated with vulnerable plaques, greater plaque burden, and adverse outcomes^6^—these results suggest that attenuated inflammation fosters a favourable environment for collateral development, whereas excessive inflammation may impede it.

In contrast to the typical M1/M2 imbalance observed in CAD adipose tissue,^7^ our data reveal a predominant M2 polarization in the PCAT of patients with well-developed collaterals. Moreover, the significant correlation between the M2/M1 ratio and microvascular density aligns with known pro-angiogenic functions of M2 macrophages,^8^ suggesting that an anti-inflammatory polarization state may enhance vascular growth within the pericoronary microenvironment.

## Study limitations

This study has several limitations. The small sample size of LAD CTO patients limits generalizability. Assessment was restricted to perivascular inflammation around the LAD without including other major vessels such as the RCA. The Rentrop classification is qualitative and lacks precision, and collateral circulation was evaluated only through this method without more detailed stratification. The definition of macrophage M1/M2 polarization relied solely on immunohistochemistry for iNOS and CD206; although widely accepted, additional markers or functional assays would strengthen the assessment. vWF staining cannot differentiate mature from immature microvessels, necessitating more accurate metrics. Finally, while M2 macrophages are associated with CCC, the specific regulatory mechanisms remain unclear and require further investigation.

## Conclusions

M2 macrophage polarization in PCAT is associated with enhanced collateralization in LAD CTO patients, supported by lower FAI, greater microvascular density, and pro-angiogenic gene expression. This implicates PCAT as a promising target for future ischaemic heart disease therapies.

## Data Availability

The datasets used and/or analysed during the current study are available from the corresponding author on reasonable request.

## References

[1] Kim EK, Choi J-H, Song YB, Hahn J-Y, Chang S-A, Park S-J, Lee S-C, Choi S-H, Choe YH, Park SW, Gwon H-C. A protective role of early collateral blood flow in patients with ST-segment elevation myocardial infarction. Am Heart J 2016;171:56–63. 26699601 10.1016/j.ahj.2015.10.016

[2] Tan N, Dey D, Marwick TH, Nerlekar N. Pericoronary adipose tissue as a marker of cardiovascular risk: JACC review topic of the week. J Am Coll Cardiol 2023;81:913–923. 36858711 10.1016/j.jacc.2022.12.021

[3] Sen F, Yilmaz S, Sen Ö, Balc KG, Duman İ, Topaloglu S, Temizhan A, Aras D. Epicardial adipose tissue is related to coronary collateral vessel formation in patients with acute coronary syndrome. Scand Cardiovasc J 2015;49:130–135. 25752649 10.3109/14017431.2015.1023345

[4] Enhos A, Sahin I, Can MM, Biter I, Dinckal MH, Serebruany V. Relation of coronary collateral circulation with epicardial fat volume in patients with stable coronary artery disease. J Geriatr Cardiol 2013;10:344–348. 24454327 10.3969/j.issn.1671-5411.2013.04.006 PMC3888916

[5] Ahmed B, Farb MG, Karki S, D'Alessandro S, Edwards NM, Gokce N. Pericardial adipose tissue thrombospondin-1 associates with antiangiogenesis in ischemic heart disease. Am J Cardiol 2024;210:201–207. 37863116 10.1016/j.amjcard.2023.09.104 PMC10842123

[6] Goeller M, Rahman Ihdayhid A, Cadet S, Lin A, Adams D, Thakur U, Yap G, Marwan M, Achenbach S, Dey D, Ko B. Pericoronary adipose tissue and quantitative global non-calcified plaque characteristics from CT angiography do not differ in matched south Asian, east Asian and European-origin Caucasian patients with stable chest pain. Eur J Radiol 2020;125:108874. 32087467 10.1016/j.ejrad.2020.108874 PMC8444621

[7] Hirata Y, Tabata M, Kurobe H, Motoki T, Akaike M, Nishio C, Higashida M, Mikasa H, Nakaya Y, Takanashi S, Igarashi T, Kitagawa T, Sata M. Coronary atherosclerosis is associated with macrophage polarization in epicardial adipose tissue. J Am Coll Cardiol 2011;58:248–255. 21737014 10.1016/j.jacc.2011.01.048

[8] la Sala A, Pontecorvo L, Agresta A, Rosano G, Stabile E. Regulation of collateral blood vessel development by the innate and adaptive immune system. Trends Mol Med 2012;18:494–501. 22818027 10.1016/j.molmed.2012.06.007

